# Phosphonoformate Crystalluria, A Warning Signal of Foscarnet-Induced Kidney Injury

**DOI:** 10.1016/j.ekir.2020.08.019

**Published:** 2020-08-22

**Authors:** Christine Deffert, Catherine Stoermann, Thomas Ernandez, Mitja Nabergoj, Yves Chalandon, Philippe Jaeger

**Affiliations:** 1 Division of Laboratory Medicine, Diagnostic Department, Geneva University Hospitals, Geneva, Switzerland; 2 Division of Laboratory Medicine, Department of Medical Specialties, Faculty of Medicine, University of Geneva, Geneva, Switzerland; 3 Division of Nephrology, University Hospital and Faculty of Medicine of Geneva, Geneva, Switzerland; 4 Division of Haematology, Department of Oncology, University Hospital and Faculty of Medicine of Geneva, Geneva, Switzerland; 5 Center for Nephrology, Royal Free, University College of London, London, UK

## Introduction

Foscarnet (pyrophosphate analog trisodium phosphonoformate) is the standard treatment of ganciclovir-resistant cytomegalovirus (CMV) infections or when administration of ganciclovir is not possible. It acts as an inhibitor of DNA polymerase and retroviral reverse transcriptase of all herpes viruses. Adverse effects are numerous and include nephrotoxicity and electrolyte disorders such as hypocalcemia.

Foscarnet has been reported to induce reversible acute kidney injury (AKI), kidney tubular necrosis, distal tubular acidosis, and nephrogenic diabetes insipidus.^1^ Reported incidence of foscarnet nephrotoxicity varies between 20% and 66%.^2^^,^^3^

Foscarnet-induced kidney injury has been largely attributed to tubulointerstitial damage,^2^^,^^4^ but crystal deposits in tubular cells or tubular lumens^5^^,^^6^ as well as in glomerular capillaries^6^^,^^7^ have been repeatedly reported, suggesting that a form of crystalline nephropathy may be another causative mechanism. Already in 1990 phosphonoformate crystals were observed within glomerular capillary lumens in kidney biopsy material from patients treated with foscarnet^6^^,^^7^ with infrared documentation of the crystals in 1998.^3^ Phosphonoformate crystals have been found deposited in various organs,^8^ mostly in the kidney, and numerous reports of crystal deposition in kidney biopsy material have been published ever since.

Until now, the relative contribution of phosphonoformate crystal deposition to foscarnet-induced nephrotoxicity has remained uncertain because patients receiving the antiviral drug suffer from severe concurrent pathologies and are often treated with other nephrotoxic drugs. Another reason for said uncertainty is that other drugs potentially leading to crystalline obstruction, such as acyclovir and indinavir, have been associated with presence of crystals in urine, whereas such was not the case for foscarnet.^9^

In this article, we describe for the first time phosphonoformate crystalluria observed in 6 cases treated with foscarnet (Table 1). These crystals have been identified both, with their specific morphology and by Fourier-transform infrared spectrometry. Crystalluria, followed by AKI were timely related to foscarnet administration.

**Table 1:** Teaching points

1. Acute kidney injury is a common complication of foscarnet therapy.
2. Foscarnet (phosphonoformate) crystals usually reported in kidney biopsy can also be detected in urine.
3. Crystalluria precedes development of foscarnet-induced kidney injury.
4. Early screening of crystalluria should be performed in patient treated with foscarnet to anticipate and may be prevent development of acute kidney injury.

## Case Presentation

### Clinical and Biological Data

Reports of 6 patients treated with foscarnet and whose urinary sediment revealed presence of phosphonoformate crystals in urinary sediments.

#### Patient 1

A 71-year-old woman diagnosed with acute myeloid leukemia underwent, while in complete remission, a nonmyeloablative allogeneic hematopoietic stem cell transplantation with HLA-compatible unrelated donor. Conditioning regimen consisted in fludarabine and total body irradiation; graft-versus-host disease (GvHD) prophylaxis consisted of mycophenolate mofetil and ciclosporine. Following transplantation, the patient presented multiple complications such as a reactivation of CMV disease (CMV serostatus: D−, R+). Ganciclovir was administered without effect on the viral load of CMV. Hence, based on *in vitro* documentation of resistance of CMV to ganciclovir, the patient was switched to foscarnet. Phosphonoformate crystalluria (proven by infrared spectrometry) was observed 14 days after the onset of foscarnet therapy and associated with a nonglomerular hematuria and renal tubular epithelial cells in urine sediment. Progressive elevation of serum creatinine concentration occurred (AKI Kidney Disease: Improving Global Outcomes [KDIGO] stage 3), despite adaptation of foscarnet doses to kidney function. The patient died after probable fungal bronchopneumonia and septicemia while still on foscarnet.

#### Patient 2

A 35-year-old man diagnosed with acute lymphoblastic leukemia achieved a second complete remission following salvage chemotherapy after a first relapse. Stem cell transplantation from an HLA-matched unrelated donor was performed following myeloablative conditioning. CMV serostatus was D−, R−. To prevent GvHD, cyclosporine with a short course of methotrexate was administered. Numerous complications occurred after transplantation, including an human herpesvirus 6 encephalitis. For the latter, ganciclovir was started and replaced after 24 hours by foscarnet because of concerns about known associated myelotoxicity with high doses of ganciclovir. Phosphonoformate crystalluria (proven by infrared spectrometry) was observed 7 days after onset of foscarnet associated with a nonglomerular hematuria but without renal tubular epithelial cells in urine sediment. Because of a stable kidney function, foscarnet administration was maintained at unchanged doses. Despite the human herpesvirus 6 encephalitis resolution, the patient died from acute respiratory failure due to uncontrolled bleeding.

#### Patient 3

A 59-year-old woman diagnosed with acute myeloid leukemia received successfully an allogeneic hematopoietic stem cell transplantation from an HLA-compatible unrelated donor following a reduced-intensity conditioning. The GvHD prophylaxis regimen was mycophenolate mofetil and cyclosporine. In the post-transplantation phase, the patient presented several complications, such as reactivation of CMV disease (CMV serostatus: D+, R+). Foscarnet was initiated and phosphonoformate crystalluria (proven by infrared spectrometry) was observed 7 days after onset of therapy associated with renal tubular epithelial cells in urine sediment. No hematuria was observed. The patient developed AKI KDIGO stage 1 24 days after foscarnet initiation.

#### Patient 4

This patient was a 63-year-old man who underwent liver transplantation for alcohol-induced cirrhosis. Despite anti-CMV (serostatus D+, R−) prophylaxis, the patient developed CMV infection against what he first received valganciclovir. The viral load of CMV further increasing, the patient was switched to foscarnet; however, phosphonoformate crystalluria (proven by infrared spectrometry) was observed 1 month after onset of the treatment with foscarnet and was accompanied by glomerular haematuria, leukocyturia, and renal tubular epithelial cells in urine sediment. The patient developed AKI KDIGO stage 1 47 days after onset of the treatment with foscarnet. CMV viral load decreased but remained present under bitherapy (foscarnet/ganciclovir, then ganciclovir/letermovir*).* Elevated creatinine persisted despite discontinuation of foscarnet.

#### Patient 5

A 73-year-old man, previously diagnosed with myelodysplastic syndrome, underwent hematopoietic stem cell transplantation from an HLA-haploidentical donor. CMV serostatus: D−, R−. GvHD prophylaxis consisted of post-transplant cyclophosphamide, tacrolimus, and mycophenolate mofetil. In the post-transplantation phase, the patient presented several complications, including oral herpes (HSV1) infection. Because of proven *in vitro* valaciclovir resistance, foscarnet was introduced: phosphonoformate crystalluria was observed at day 11 after onset of therapy. Neither red blood cells nor any other cells were seen in the urine sediment. The patient developed AKI KDIGO stage 2 at day 12. The viral infection was effectively treated, but the patient died 2 months later due to an invasive fungal infection.

#### Patient 6

This patient was a 70-year-old woman, diagnosed with high-grade myelodysplastic syndrome. After having achieved remission with systemic chemotherapy, she underwent a haploidentical stem cell transplantation. For GvHD prevention, she received post-transplant cyclophosphamide, tacrolimus, and mycophenolate mofetil. CMV serostatus: D−, R+. Despite anti-CMV prophylaxis with letermovir, CMV viral load increased from month 3 on, and a preemptive treatment with valganciclovir was introduced. Given a further increment of the viral load despite this treatment, foscarnet was subsequently started. Phosphonoformate crystalluria (proven by infrared spectrometry) was observed at day 9 after onset of foscarnet associated with a nonglomerular hematuria and renal tubular epithelial cells in urine sediment. The patient developed AKI KDIGO stage 1 at day 14.

### Crystalluria

**Figure 1:**
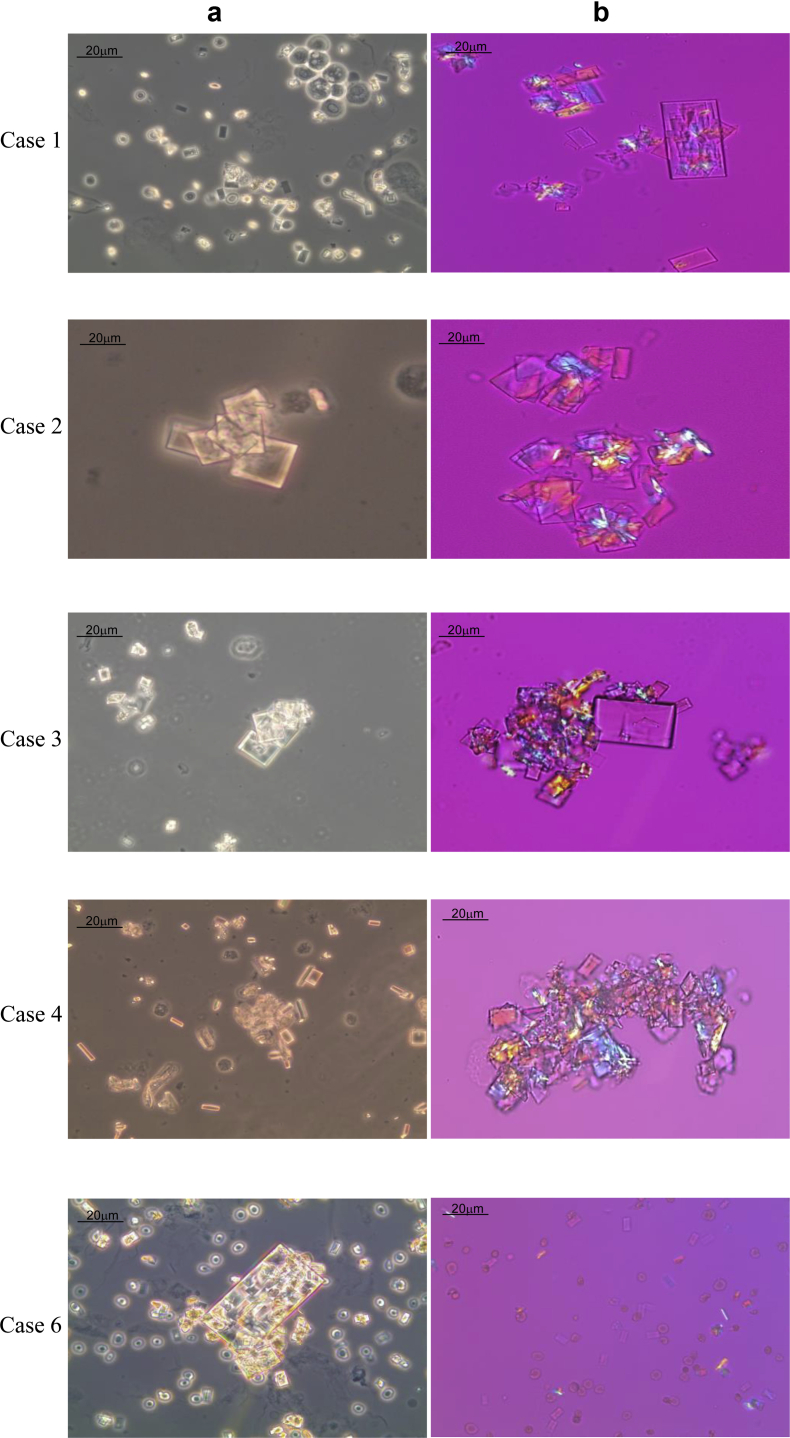
Foscarnet crystals in urinary sediment. Phosphonoformate crystals visualized by contrast phase microscopy (a) and polarized light with compensatory lens (b). Original magnification ×400.

Phosphonoformate crystals exhibited rectangular and square shapes, sometimes shapes of a thin stick when they were positioned on the edge. They often formed large aggregates. Their sizes varied from 16 to 70 μm (Figure 1). Single crystals did not polarize; they only did so when present as aggregates. The number of crystals varied greatly from patient to patient and even between samples from the same patient. pH of urine containing crystals ranged from 5.5 to 7.5.

### Urinary Crystal Identification

**Figure 2:**
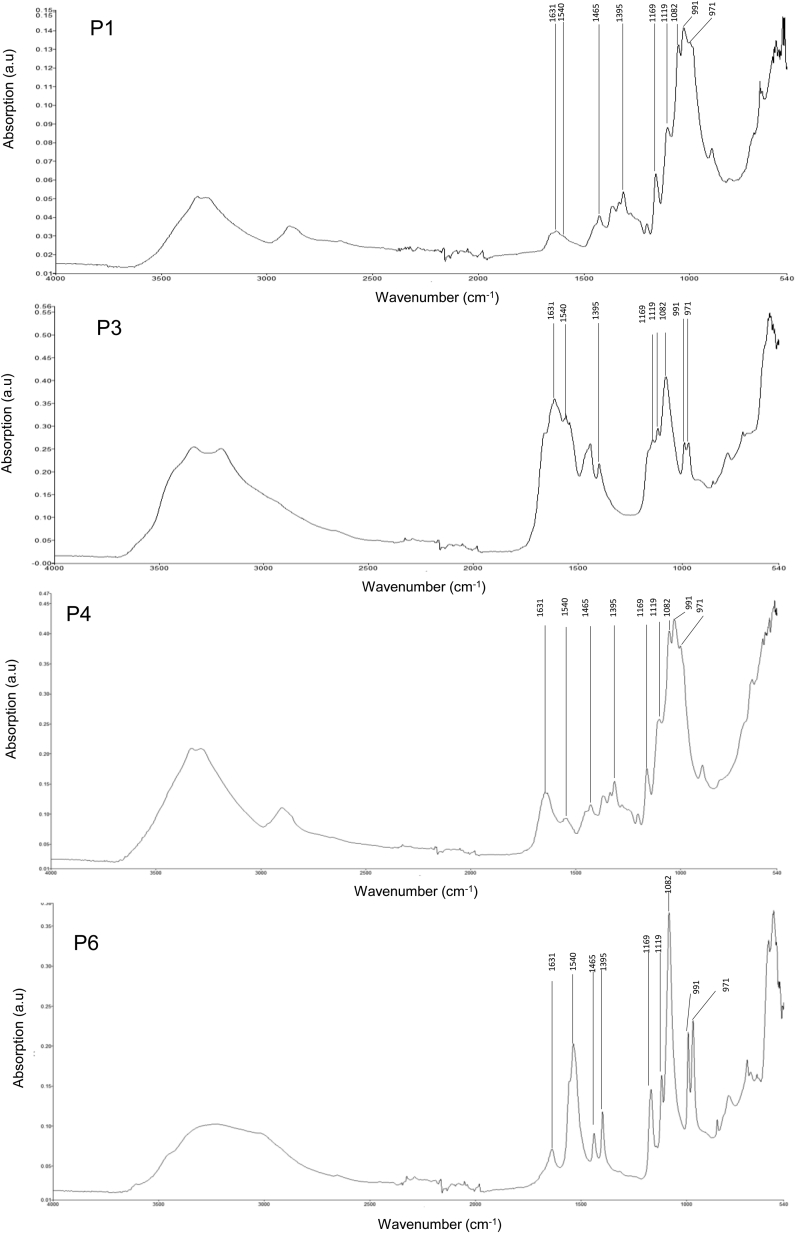
Infrared (IR) spectra of the crystals found in urine of 4 patients. IR analysis was performed in urine from 4 cases (patients 1, 3, 4, and 6). Four IR spectra of urinary crystals from each patient had typical IR bands of foscarnet: 1540 and 1395 cm^−1^ (carboxylate domain) and 1169, 1119, and 1082 cm^−1^ (phosphate domain). Spectra included 2 more bands at 971 and 991 cm^−1^. P, patient.

All analyzed Fourier-transform infrared (FTIR) spectra of urinary crystals had typical infrared (IR) bands of foscarnet: 1540 and 1395 cm^−1^ (carboxylate domain) and 1169, 1119, and 1082 cm^−1^ (phosphate domain) (Figure 2). These FTIR measurements confirmed that the crystals observed in those urine sediments were made of phosphonoformate.

The 4 urine specimens analyzed by IR spectrometry displayed differences in intensity between bands 971 and 991 cm^−1^. The band at 991 cm^−1^ was higher than that at 971 cm^−1^ in patients 1 and 4, but for patients 3 and 6, band intensity at 971 and 991 were equal. Thus, the proportion of both salts in the mix varied from one patient to another.

### Acute Kidney Injury

**Figure 3:**
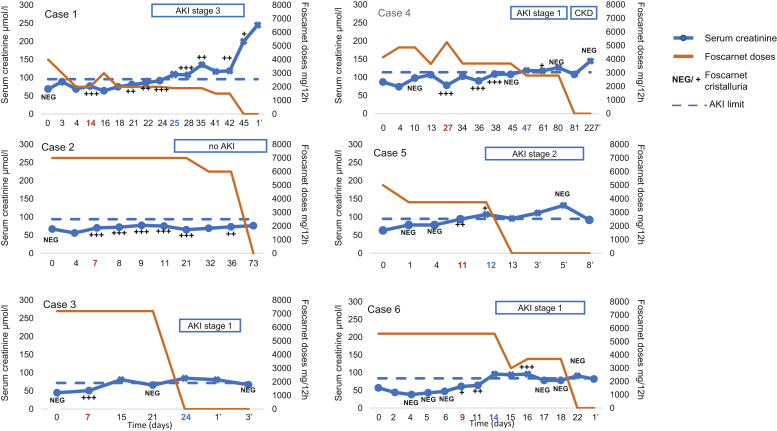
Time course of foscarnet crystalluria and kidney injury. Relevant parameters, such as phosphonoformate crystalluria, serum creatinine, and foscarnet doses, were described for the 6 patients. Foscarnet therapy began at day 0 and has been given twice daily. The symbol ' corresponds to the day after stop of the foscarnet therapy. Days in red correspond to days when foscarnet crystalluria appeared and days in blue correspond to days when acute kidney failure (AKI) appeared. The blue line corresponds to AKI defined by Kidney Disease: Improving Global Outcomes (KDIGO) criteria. Serum creatinine value is represented by a dot when the value is under this AKI limit and represented by a cross when the value is above this AKI limit. The semi-quantitative determination of crystals was defined as: +, 1 per 3 high-power fields; ++, 2–4 per high-power fields; and +++, more than 4 per high-power fields.

Among the 6 patients treated with foscarnet and who presented with phosphonoformate crystalluria, 5 of them further developed a kidney injury (Figure 3). Patient 1 developed AKI KDIGO stage 3; patient 5 stage 2; and patients 3, 4, and 6 stage 1. After onset of foscarnet therapy, the median time lag for phosphonoformate crystalluria to appear was 11 days (range 7 to 27 days), whereas that for AKI to occur was 24 days (range 12 to 47 days). Occurrence of AKI always followed appearance of crystalluria by a median time lag of 11 days. Patients with crystalluria presented diluted urine as estimated by specific gravity ranging from 1.004 to 1.014. Among the 5 patients with AKI, 4 of them showed in their urine sediment crystalluria and leukocyturia and/or renal tubular epithelial cells. None of them showed casts.

As serum creatinine level increased, the amount of crystals in urine decreased (median serum creatinine was 112.5 μmol/l when crystal quantities were estimated at +, median serum creatinine was 86 μmol/l when crystal quantities were estimated at ++, and finally median serum creatinine was 77 μmol/l when crystal quantities were estimated at +++). The semi-quantitative determination of crystals was defined as: +, 1 per 3 high-power fields; ++, 2–4 per high-power fields; and +++, more than 4 per high-power fields defined in Supplementary Methods.

The higher the dose of foscarnet administered, the earlier phosphonoformate crystalluria appeared. The 3 patients (patients 2, 3, and 6) who received doses of more than 10 g per day were also those who had the earliest crystalluria, that is, fewer than 10 days after the onset of treatment. On the other hand, the early onset of AKI was not related to high doses of foscarnet (Figure 3).

Reduction of foscarnet doses was not associated with any decline in serum creatinine concentrations. Only withdrawal of treatment after short-term treatment (<20 days) allowed reversibility of kidney impairment (patients 3, 5, and 6).

## Discussion

We report the first identification of phosphonoformate crystals in urine of patients treated with foscarnet, and describe the time lag between appearance of crystalluria and AKI known to occur in the context of that therapy.

Phosphonoformate crystals in material obtained from kidney biopsies have been described as “short polarized sticks with angular edges.”^1^^,^^2^ Urine crystals had a similar shape but did not polarize as single crystals, whereas they did so when aggregated in clusters as it occurs in the kidney issue. Because phosphonoformate crystals had never been reported in urine before, identification by IR spectroscopy in urine sediments was required to confirm their chemical composition. We found the typical bands previously reported,^2^^,^^3^^,^S1 confirming that the observed crystals were indeed made of phosphonoformate.

Phosphonoformate may crystallize as calcium salt or as sodium salt.^2^^,^^3^ Based on *in vitro* experiments, calcium salt is less soluble in urine than sodium salt of phosphonoformic acid. In this report, we have found a mix of sodium and calcium salts of phosphonoformic acid crystallized in urine. On the other hand, we have not found crystals corresponding to trisodium phosphonoformate. Patients 3 and 6 displayed an equal proportion of sodium and of calcium salts, as opposed to the other 2 patients (1 and 4) for which IR band intensity of calcium salts (991 cm^−1^) were dominant compared with those of sodium salts (971 cm^−1^). Patients 3 and 6 were also patients with few episodes of crystalluria and who only developed AKI stage 1 as opposed to other patients (stage 3 and 2, respectively, for patient 1 and 4). An equal proportion of sodium and calcium salts might have attenuated the trend for phosphonoformic acid to crystallize, and thereby the potential severity of kidney injury. If this were true, the spectrum of the crystals and the salt proportion of both salts at IR of urine sediments might turn out to help and predict the degree of severity of kidney damage.

Numerous risk factors of drug crystallization have been recognized so farS2: they may be related to the drug (dose, duration of treatment, solubility, simultaneous use of other molecules) or related to the patient (urinary pH, urine specific gravity, or stasis).^4^ Overall, we have not identified risk factors for foscarnet crystallization in urine (Figure 3). Patients reported to have developed AKI after foscarnet infusion classically received repeated and/or high doses of the drug (i.e., 6 to 12 g per day).^4^ Our patients who developed crystalluria had received foscarnet doses ranging from 3 to 14 g per day; however, severity of crystalluria or of AKI did not appear to be related to the actual dose of foscarnet given. On the other hand, the 3 patients who developed a rapid onset of phosphonoformate crystalluria (7 and 9 days) had received daily doses of more than than 10 g.

Duration of treatment seems to have an effect on the reversibility of the kidney damage. Indeed, the 3 patients (3, 5, and 6) with the shortest treatment time presented a rapidly reversible AKI.

Infusion of large volumes of isotonic saline, 1.5 to 2.5 liters per day, has been demonstrated to reduce kidney toxicity of foscarnet by decreasing the urinary concentration of the drug.^4^, ^5^, ^6^ Nevertheless, despite an appropriate hydration as illustrated by their very low urine specific gravity, patients presented phosphonoformate crystals in their urine. In urine with phosphonoformate crystals, the spectrum of pH ranged from 5.5 to 7.5 (data not shown). Crystallization of foscarnet does not seem to depend on urine pH, and alkalinization of urine is probably of no help for crystal solubilization. The occurrence of foscarnet crystallization did not show differences between acidic or alkaline urine. Finally, foscarnet nephrotoxicity might be amplified by concurrent administration of other nephrotoxic drugs. In our report, one patient simultaneously received cyclosporine and foscarnet (patient 2) but he was also the only one whose serum creatinine concentration remained stable.

Presence of crystalluria is a sign of supersaturation of urine and does not necessarily imply that associated-AKI is due to crystalline nephropathy. In absence of kidney biopsy, only crystalline casts in urine sediment would suggest such a causal link. Unfortunately, crystalline casts are rarely found in urine. On the other hand, white blood cells, renal tubular epithelial cells, or casts in sediment-associated crystalluria indicate also intrarenal crystal-related injury.S4 In analyzed urine sediment, 4 patients showed white blood cells or renal tubular cells enhancing the fact that crystalluria might cause kidney injury. The patient who did not develop AKI did not have white blood cells or renal tubular epithelial cells in urine sediment.

Presence of foscarnet (phosphonoformate) crystals in urine may be a predictive factor of crystalline nephropathy. However, 5 of the 6 cases received allogeneic hematopoietic stem cell transplantation and presented with variable degrees of thrombocytopenia preventing kidney biopsy to determine the underlying cause of AKI. The discovery of phosphonoformate crystals in urine allows, in these cases, to strongly suspect foscarnet nephrotoxicity without invasive procedure.

This observation raises new questions, such as the following.

• Why is it the first time that phosphonoformate crystalluria is described in patients on foscarnet therapy, whereas phosphonoformate crystals have been known for years to be deposited in various organs of patients treated with that drug.^7^ Maybe early check for crystals in urine has never been considered before; or maybe at the time kidney injury occurs tubular obstruction prevents further release of crystals in urine. We have presented some evidence to support this latter hypothesis: crystalluria always appeared ahead of kidney injury onset; the median time lag for phosphonoformate crystalluria to appear was 11 days, whereas that for AKI to occur was 24 days. Occurrence of AKI always followed appearance of crystalluria by a median time lag of 11 days. On the other hand, serum creatinine level increased when the amount of crystals in urine decreased.
• Where do the crystals actually first form? Is it in the glomerular urinary space or in a specific segment of the kidney tubules? This would need to be reconciled with previous observations of phosphonoformate crystal deposition in the lumen of glomerular capillaries.

## Conclusions

The present report highlights the fact that phosphonoformate crystals in urine of patients treated with foscarnet may be taken as a warning signal of imminent development of AKI induced by the drug. This should trigger preemptive measures, such as hyperhydration, dose down-titration, or consideration of an alternative regimen, although conclusive evidence of the effectiveness of those actions is lacking.

## Disclosure

All the authors declared no competing interests.

## Footnotes

Supplementary File (PDF) **Supplementary Methods**. **Supplementary References**.
