# Supplementary material for: Phosphonoformate Crystalluria, A Warning Signal of Foscarnet-Induced Kidney Injury
Source: Kidney Int Rep. 2020 Aug 22;5(11):2102–8. doi: 10.1016/j.ekir.2020.08.019 (PMC7609899; doi:10.1016/j.ekir.2020.08.019)
Supplement: Supplementary File (PDF) [file mmc1.pdf]

SUPPLEMENTARY METHODS

Dipstick analyses

Urine samples were kept at room temperature and rapidly processed. Urine pH and specific gravity were determined from dipstick using UX-2000 analyser (Sysmex®). Dipstick analysis was also used for semi-quantification of hemoglobin, protein, leukocyte esterase, nitrites, urobilinogen, bilirubin, glucose and ketone bodies. White (WBC) and red blood cells (RBC) were quantified by flow cytometry with the automated urine particle analyser UF-1000i (Sysmex). Erythrocyte morphology was determined by phase contrast microscopy performed in urine sediments.

Microscopic examination of crystals

As stated above, urine samples were kept at room temperature and rapidly processed. Urines were centrifuged (600 G, 5 min) to be concentrated. Obtained sediments were examined by phase contrast microscopy and polarized light with a compensatory lens at x400 magnification. The compensatory lens allows polarized and no polarized crystals to be visualized at the same time.

The semi-quantitative determination of crystals was defined as: +, 1 per 3 high-power fields (HPF); ++, 2-4 per HPF; +++, more than 4 per HPF.

Infrared analyses of crystals

The microscopic examinations were combined with Fourier-transform infrared (FTIR) spectrometry to confirm the nature of unusual crystals. For infrared analysis, urine was filtered using a Whatman 542 filter (Springfield Mill, Maidstone, Kent, UK), and the retained sediment left to dry out for a few hours at room temperature. The filter was analysed by FTIR spectrometry (Spectrum 100, Perkin-Elmer®). The spectra generated from analyses of the crystals were identified by comparison with reference infrared spectra of pure compounds.

Renal function estimation

Serum creatinine concentrations were measured according to Jaffé et al (COBAS 8000 Roche®). Normal values are 62 to 106 µmol/L for men and 44 to 80 µmol/L for women. AKI was defined according to Kidney Disease Improving Global Outcomes (KDIGO) criteria [S3] : Stage 1 : increase in serum creatinine by ≥ 0.3 mg/dl (≥ 26.5 µmol/l) within 48 hours; or increase up to ≥ 1.5-1.9 fold baseline, Stage 2 : increase in serum creatinine to ≥ 2 -2.9 fold baseline and Stage 3 : increase in serum creatinine by ≥ 354 µmol/l within 48 hours, or increase up to ≥ 3 fold baseline. The baseline was the value of serum creatinine concentration on the day of the first injection of foscarnet.

SUPPLEMENTARY REFERENCES

S1. Zanetta G, Maurice-Estepa L, Mousson C, *et al.* Foscarnet-induced crystalline glomerulonephritis with nephrotic syndrome and acute renal failure after kidney transplantation. *Transplantation* 1999; **67:** 1376-1378.

S2. Daudon M, Frochot V, Bazin D, *et al.* Drug-Induced Kidney Stones and Crystalline Nephropathy: Pathophysiology, Prevention and Treatment. *Drugs* 2018; **78:** 163-201.

S3. Kellum JA, Lameire N, Group KAGW. Diagnosis, evaluation, and management of acute kidney injury: a KDIGO summary (Part 1). *Critical care.* 2013;17(1): 204.

S4. Cavanaugh C, Perazella MA. Urine Sediment Examination in the Diagnosis and Management of Kidney Disease: Core Curriculum 2019. *American journal of kidney diseases : the official journal of the National Kidney Foundation* 2019; **73:** 258-272.
